# Supplementary material for: The Impact of Controlled Fermentation Temperature on Chemical Composition and Sensory Properties of Cacao
Source: Foods. 2025 Apr 22;14(9):1441. doi: 10.3390/foods14091441 (PMC12071279; doi:10.3390/foods14091441)
Supplement: Supplementary file 1 [file foods-14-01441-s001.zip › foods-3538972-supplementary.pdf]

S1. Photographs of the method.

a) Bean extraction was carried out manually, the wet cocoa was placed in plastic containers and then placed in stainless steel tanks.

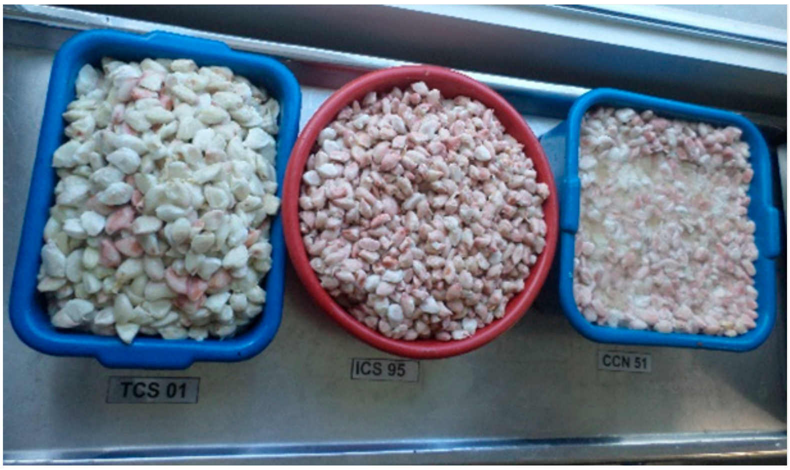

Photo: Lucero Gertrudis Rodriguez Silva

b) The fermentation process was carried out inside the incubator

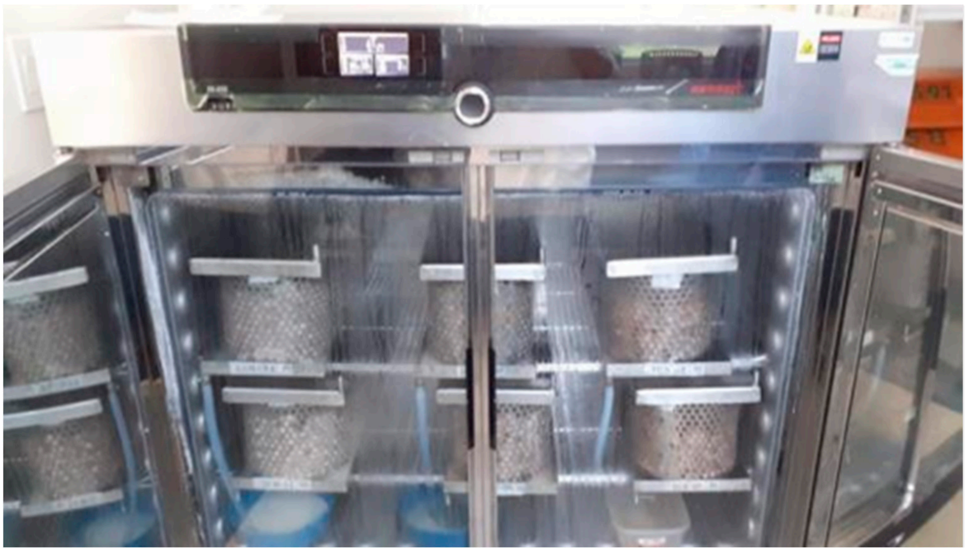

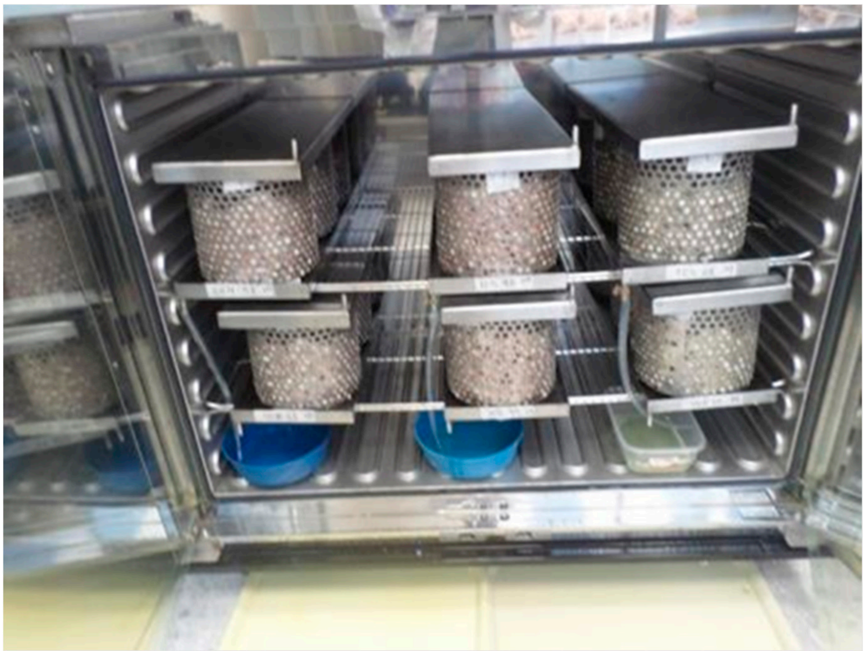

Photos: Lucero Gertrudis Rodriguez Silva

c) *Photograph of the fermented bean on the fifth day*

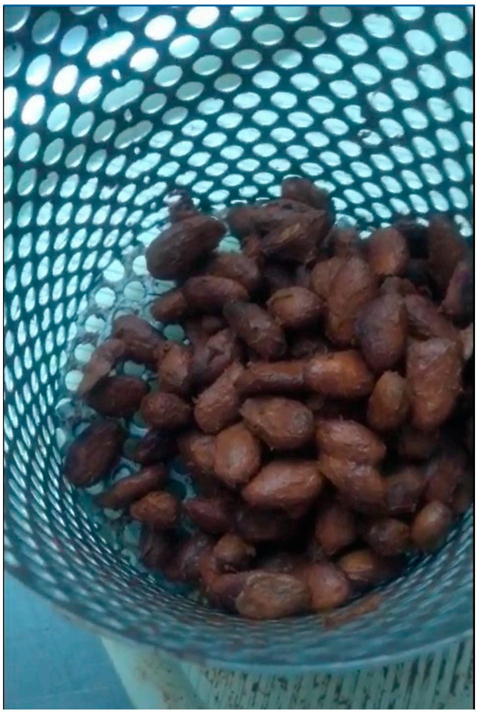

Photo: Lucero Gertrudis Rodriguez Silva
